# An Evidence-Based IT Program With Chatbot to Support Caregiving and Clinical Care for People With Dementia: The CareHeroes Development and Usability Pilot

**DOI:** 10.2196/57308

**Published:** 2024-12-23

**Authors:** Nicole Ruggiano, Ellen Leslie Brown, Peter J Clarke, Vagelis Hristidis, Lisa Roberts, Carmen Victoria Framil Suarez, Sai Chaithra Allala, Shannon Hurley, Chrystine Kopcsik, Jane Daquin, Hamilton Chevez, Raymond Chang-Lau, Marc Agronin, David S Geldmacher

**Affiliations:** 1 School of Social Work, University of Alabama, Tuscaloosa, AL, United States; 2 Alzheimer's Disease Research Center, University of Alabama at Birmingham, Birmingham, AL, United States; 3 Nicole Wertheim College of Nursing and Health Sciences, Florida International University, Miami, FL, United States; 4 Knight Foundation School of Computing and Information Sciences, Florida International University, Miami, FL, United States; 5 Department of Computer Science and Engineering, University of California, Riverside, Riverside, CA, United States; 6 College of Medicine, Florida State University, Tallahassee, FL, United States; 7 MIND Institute and Behavioral Health, Miami Jewish Health, Miami, FL, United States; 8 Department of Criminal Justice and Criminology, Beto Criminal Justice Center C105, Sam Houston State University, Huntsville, TX, 77341, United States; 9 Department of Neurology, Heersink School of Medicine, University of Alabama at Birmingham, Birmingham, AL, United States

**Keywords:** Alzheimer disease, artificial intelligence, caregivers, chatbot, dementia, mobile applications, conversational agent, design, apps

## Abstract

**Background:**

There are numerous communication barriers between family caregivers and providers of people living with dementia, which can pose challenges to caregiving and clinical decision-making. To address these barriers, a new web and mobile-enabled app, called CareHeroes, was developed, which promotes the collection and secured sharing of clinical information between caregivers and providers. It also provides caregiver support and education.

**Objective:**

The primary study objective was to examine whether dementia caregivers would use CareHeroes as an adjunct to care and gather psychosocial data from those who used the app.

**Methods:**

This paper presents the implementation process used to integrate CareHeroes into clinical care at 2 memory clinics and preliminary outcome evaluation. Family caregivers receiving services at clinics were asked to use the app for a 12-month period to collect, track, and share clinical information with the care recipient’s provider. They also used it to assess their own mental health symptoms. Psychosocial outcomes were assessed through telephone interviews and user data were collected by the app.

**Results:**

A total of 21 caregivers enrolled in the pilot study across the 2 memory clinics. Usage data indicated that caregivers used many of the features in the CareHeroes app, though the chatbot was the most frequently used feature. Outcome data indicated that caregivers’ depression was lower at 3-month follow-up (*t*_11_=2.03, *P*=.03).

**Conclusions:**

Recruitment and retention of the pilot study were impacted by COVID-19 restrictions, and therefore more testing is needed with a larger sample to determine the potential impact of CareHeroes on caregivers’ mental health. Despite this limitation, the pilot study demonstrated that integrating a new supportive app for caregivers as an adjunct to clinical dementia care is feasible. Implications for future technology intervention development, implementation planning, and testing for caregivers of people living with dementia are discussed.

## Introduction

### Overview

Caregiving and clinical care for the 7 million Americans with Alzheimer disease or a related dementia (ADRD) is complex and expensive, with projected medical costs for this population increasing to just under $1 trillion in 2050 [1]. Further complicating care for people living with dementia are communication barriers among patients, their family caregivers (eg, spouses and adult children), and providers (eg, physicians and nurses), which may negatively impact the collection of information that is needed to make clinical decisions and create additional challenges for caregivers [2,3]. Health IT (HIT) interventions have been identified as having potential for overcoming these communication challenges [4,5]. While there has been an increase in the development and testing of HIT for this purpose, little remains known about how to effectively integrate new HIT into practice to support provider-caregiver communication. In this study, a multidisciplinary team developed a new multifunctional application, called CareHeroes, and conducted a pilot study to evaluate usage and preliminary outcomes for caregivers receiving services at 2 memory clinics. This paper presents findings from the project as well as the extensive implementation process that the team underwent to develop and integrate the app into care. Implications for technology development and testing in dementia care are discussed.

### Caregiver-Provider Communication in Dementia Care

Prior research has found that some barriers to caregiver-provider communication stem from caregivers, who have reported that they sometimes have difficulty remembering all of the information they planned to discuss with the care recipient’s provider or they become too upset during the medical visit to adequately share information [6]. Other research has reported that caregivers with lower levels of health literacy or who experience anxiety when discussing difficult topics may not effectively communicate with providers [7]. There are also communication barriers stemming from providers and service systems. For example, lack of time to collect sufficient information during the medical visit, insufficient sharing of clinical information across care settings [8], and lack of access to community-based resources for dementia care [9,10].

Poor communication between caregivers and providers may contribute to a number of negative outcomes. For caregivers, the difficulties of health management for people living with dementia place them at increased risk of poor mental and physical health [1]. Communication challenges can also impede clinical decision-making by providers, which can have a negative impact on patient care and outcomes [10]. There are also disparities in experiences with health care providers. For example, African American caregivers are more likely than white caregivers to report that they lacked knowledge about ADRD before their loved one’s diagnosis and greater dissatisfaction with the interactions they have with providers regarding dementia care [11], which contribute to disparities in outcomes. For example, while African American and Latino/a populations are at higher risk of ADRD compared to their non-Latino/a White counterparts, they are more likely to go undiagnosed or misdiagnosed [12,13]. Caregivers living in rural communities may face additional barriers to interacting with providers due to the inaccessibility of services [14].

### Opportunities and Limitations of IT and AI Apps for Dementia Caregiving and Care

Over the past decade, there has been increased attention on how IT and artificial intelligence (AI) may be used to overcome challenges in dementia care and caregiving [15,16]. An increasing number of studies have examined how tech-based platforms may support caregivers of people living with dementia, though many of the interventions that have been reported in the research literature have not reflected advancements in technology, such as smartphone apps or AI tools [15]. For commercially available smartphone apps targeting caregivers, reviews of existing technologies have shown that they are limited in function, compared to the demands of caregiving; are underresearched, especially with underserved populations; and are not designed to be integrated within clinical care settings [15-17]. Hence, there is a need for increased research that develops an evidence base for advanced technology tools that support this population.

### Developing the CareHeroes App

In response, our interdisciplinary team (social work, nursing, physical therapy, geriatric psychiatry, neurology, and computer science) developed CareHeroes, a web-based application that was designed with ongoing input from caregivers and providers, including those from underserved populations [18]. CareHeroes supports caregiver-provider communication through several functions. First, it supports caregiver health literacy through text and video caregiver educational resources about dementia and caregiving. It also supports education through an educational chatbot. CareHeroes also improves the collection and sharing of clinical information about the care recipient between the caregiver and provider. The app includes validated clinical assessment tools, such as the Revised Memory and Behavioral Problem Checklist [19] and ADL/IADL assessments [20], that the caregiver can complete in real time, and the algorithm can create a color-coded table of the responses that can be tracked over time and shared with the provider.

CareHeroes also supports caregivers through self-assessments of depression (Patient Health Questionnaire for Depression and Anxiety (PHQ-2) [21], burden (Zarit Caregiver Burden Inventory) [22], and positive aspects of caregiving [23] and provides the caregiver with feedback based on the results of their responses. A full description of CareHeroes, its design, and features has been reported elsewhere [18,24]. All features on CareHeroes, except for the chatbot, are available in Spanish [25]. There are future plans to translate the chatbot into Spanish.

#### Preliminary Work to Maximize Potential Adoption in Real-World Settings

The ultimate goal for CareHeroes was to develop an app that could be integrated into clinical care in real-world settings, and therefore the research team took several initial steps in developing the app to maximize its potential for adoption by providers and caregivers as end users [26]. Early on, the research team conducted needs assessments with stakeholders, which identified potential features of CareHeroes that caregivers and providers though would be beneficial. Surveys were conducted with geriatric home care workers to learn more about communication needs in dementia care [27] and late-life depression care [28]. These studies found that there was little communication between in-home care and primary care providers about older patients’ mental health and that home care workers’ observations about patients’ mental health are not easily shared with other providers. The team also analyzed HIT policy for its potential impact on the app and identified logical ways of fostering interoperability and security for information sharing [29].

After developing an initial prototype of the app, the interdisciplinary team conducted a small beta-testing study in Miami, Florida, where triads of caregivers, geriatric home care managers, and primary care providers used CareHeroes for 11 weeks [18]. The study determined that it was feasible to share information through CareHeroes and that caregivers found it useful and easy to use, though it was found that adoption of the app in real-world settings would be more feasible for dyads of caregivers and primary care providers without the involvement of home health care because it reduced the administrative barriers associated with. These findings informed revisions of the app code. This revised version then underwent alpha testing with 36 caregivers, most of whom were from caregiver populations that have historically been underserved by HIT (eg, African Americans, rural dwelling) [6,30]. The team also conducted focus groups with staff at 2 collaborating memory clinics [31] to gain feedback on the design, capacity, and potential adoption of CareHeroes. This helped the team develop a clinical workflow plan (eg, which clinic staff member collects information caregivers enter in the app, deciding how the information gets to the patient’s record). The team also worked with HIT security administrators at both clinics to make sure that information sharing followed HIPAA (Health Insurance Portability and Accountability Act) and organizational policies.

#### CareHeroes Code Development

**Figure 1.**
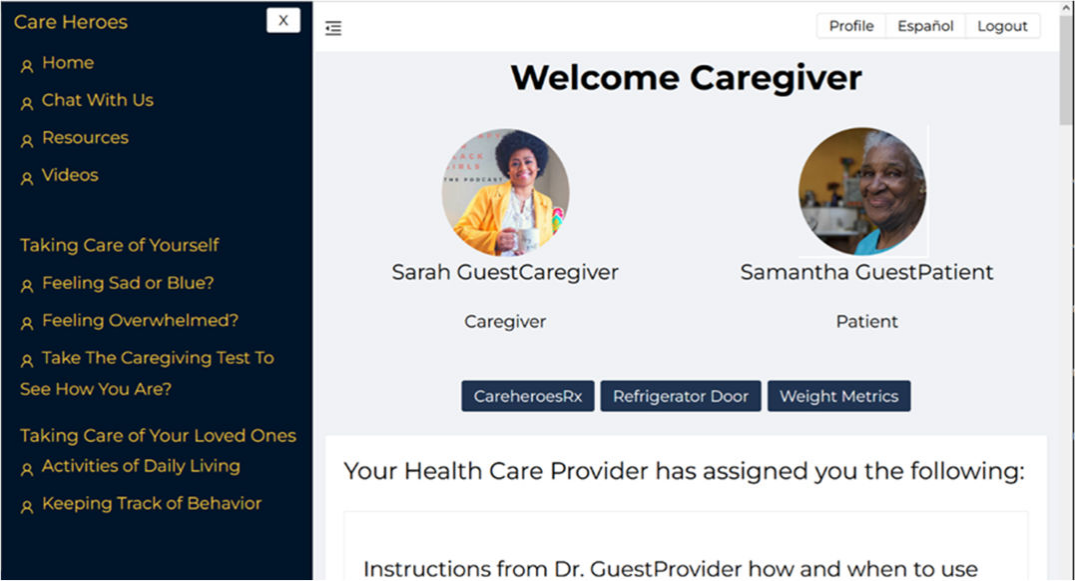
CareHeroes home page for the caregiver.

The CareHeroes application was developed using a hybrid of agile and unified software development processes [32]. Figure 1 shows the caregiver’s home page in the CareHeroes application. CareHeroes was developed based on the requirements of 3 stakeholders. These stakeholders include the health care provider, the caregiver, and the people living with dementia. The requirements included 32 use cases, with 17 caregiver use cases, 5 provider use cases, and 9 use cases focusing on the security of the system. The CareHeroes architectural design is based on a 4-tiered architecture consisting of a client tier (web-based and mobile—to be implemented), a presentation layer, a business logic layer, and the data store layer. The database structure consists of 5 parts. One part each for the user, survey, prescription, and reminder services. The final part of the database is the correlation database, which stores the keys needed to encrypt and decrypt the protected information of people living with dementia.

The implementation of CareHeroes security protocols follows the HIPAA security rule as specified in the Federal Register [33]. CareHeroes uses a role-based authentication system to ensure that only certain users with specific roles can perform certain operations within the system. All electronic communication with the CareHeroes system is encrypted, and all HTTP requests are encrypted using the Secure Sockets Layer protocol [34]. The security protocol is implemented using the database structure previously described.

#### Chatbot Programming

**Figure 2.**
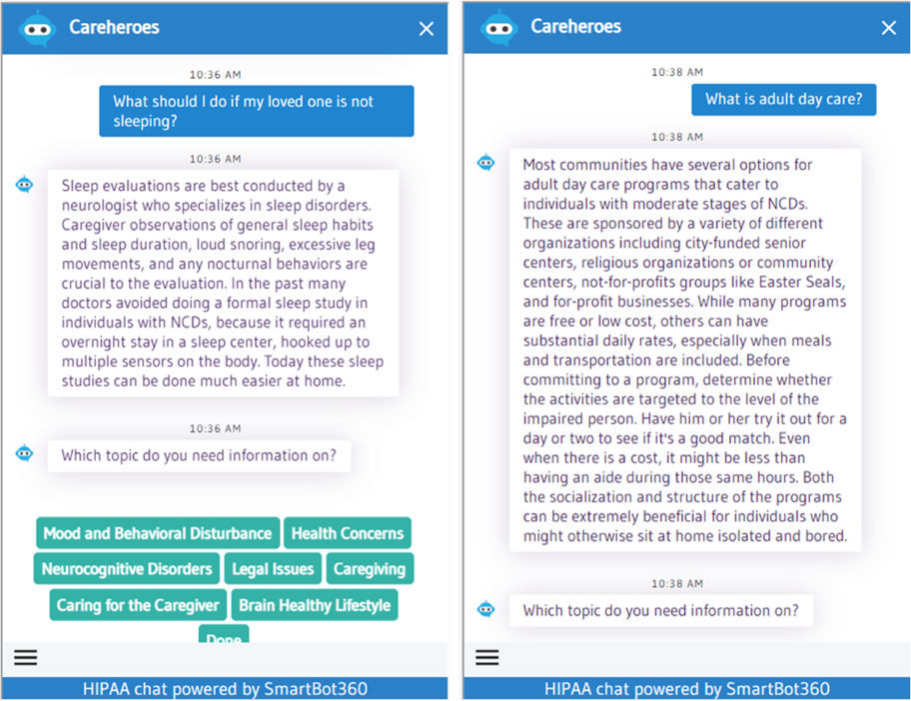
Screenshots of chatbot on the CareHeroes platform demonstrating its answers to potential caregiver questions. HIPAA: Health Insurance Portability and Accountability Act.

One of the more novel features of CareHeroes. Compared to existing apps for caregivers of people living with dementia, this is an educational chatbot. For Carehereos, the chatbot was programmed to respond to predetermined topics selected by the user (eg, mood and behavioral disturbances) and to recognized text from user questions (eg, How do I help my father eat?). The topics and content that were programmed into the chatbot and the responses it provided to users were from the book, *The Dementia Caregiver: A Guide to Caring for Someone with Alzheimer’s Disease and Other Neurocogntive Disorders* [35] by geriatric psychiatrist, Dr Marc E. Agronin. The book’s focus is guiding caregivers through caring for the people living with dementia and themselves. Minor edits were made to simplify language and to shorten responses. See Figure 2 for screenshots of the CareHeroes Chatbot with example text.

Chatbots are programmed to respond to user input (eg, questions, comments) with text or verbal language responses and have become increasingly popular in health care, especially for patient education in other health care settings [36]. For *CareHeroes*, the chatbot was programmed to respond to predetermined topics selected by the user (eg, mood and behavioral disturbances) and to recognize text from user questions (eg, How do I help my father eat?). Specifically, the user could first pick a topic, and then type in free text a question. The chatbot uses text embedding methods to perform semantic matching between the user’s question and the list of stored questions.

## Methods

### Recruitment

CareHeroes was implemented at 2 memory clinics: (1) The Frank C. and Lynn Scaduto MIND Institute at Miami Jewish Health in Miami, Florida, and (2) the Memory Disorder Clinic at the University of Alabama at Birmingham in Birmingham, Alabama. Both clinics provide a variety of services to people living with dementia and their caregivers, many of which are from underserved communities (eg, African American, Latino/a, rural dwelling). IRB approval was obtained from all institutions involved in this project before initiating the study. Recruitment took place between February 2021 and April 2022. In most cases, caregivers were recruited during telehealth visits with providers at either clinic. To be eligible for the study, caregivers had to: have regular access to the internet (via computer or smartphone); be 21 years old or older; provide caregiving activities for at least 2 hours or more per day on average of direct assistance or supervision for a person living with dementia; and have the ability to speak and understand English or Spanish. During the telehealth session, the provider would tell the caregiver about the study and ask if they were interested in more information about the project. In addition, clinic staff reached out to caregivers who were listed on existing caregiver support groups and research participant pools to inform them about the study. Those who reported interest in the study were referred to the research team, which would follow up with the caregiver to determine eligibility and enroll those who were eligible and interested in participating in the study.

### Data Collection

#### Interview Data and Measures

The data collection plan included telephone or Zoom interviews with caregivers at baseline, 3-month, 6-month, and 12-month time points. Each interview lasted between 45 and 60 minutes. The interviewer would record responses using a Qualtrics form. Interview guides included several validated psychosocial measures, and the baseline interview included a demographic questionnaire. To minimize burden, some measures were only scheduled to be included in the baseline and 12-month follow-up interviews.

#### Communication With Providers

A 3-item scale developed by Lorig et al [37] was used to measure caregiver communication with providers. Each item used a 6-point scale (0=never to 5=always) and measures frequency of which the respondent prepares a list of questions for a physician; asks questions regarding the physician and regarding things the respondent does not know; and discusses personal problems related to the illness with a physician.

#### Depression

The PHQ-4 is a brief 4-item validated tool to assess overall psychological distress and screen for both anxiety and depressive disorders [21]. The measure includes the 2-item depression screen (PHQ-2) and the 2-item screen for anxiety disorders (Generalized Anxiety Disorder 2 item [GAD-2]). The 4-item response options are scored from 0 to 3, 0=not at all, 1=several days, 2=more than half the day, and 3=nearly every day. The total PHQ-4 score is computed by adding together the 4 items; the overall psychological distress score range is from 0‐12 (normal: 0‐2; mild: 3‐5; moderate: 6‐8; severe 9‐12). A total score for the 2 depression items (depressed mood, anhedonia) of 3 or greater indicates depression and a total score of 3 or more for the 2 anxiety items (feeling anxious, uncontrolled worry) indicates anxiety. The PHQ-4 properties have been established for Spanish-speaking Hispanic Americans [38].

#### Health Literacy About Alzheimer Disease

The Alzheimer’s Disease Knowledge Scale (ADKS) is a validated 30-item true or false tool that is used to evaluate the participants knowledge on aspects of Alzheimer disease [39]. This short survey takes approximately 5‐10 minutes to complete, where each item includes a statement about Alzheimer disease, including risk factors, diagnosis, assessment and symptoms, prognosis, effects on caregiving, and management of the disease.

#### Caregiver Burden

The Zarit Burden Interview used is a 22-item survey to collect information on caregiver burden, including items relating to dependence in activities of daily living, and assess the frequency of any problem behaviors [40]. The items are answered as never (0), rarely (1), sometimes (2), quite frequently (3), or nearly always (4). A total score ranges 0‐88, with 0‐21 identified as no to mild burden, 21‐40 as mild to moderate burden, 41‐60 as moderate to severe burden, and scores 61 or above identified as severe burden.

#### User Data

CareHeroes was also programmed to automatically collect anonymized user data from the app. The program tracked several activities for those using the app, including (1) dates and times for Log-in and log-out; (2) dates and times for clicks on links for educational resources and videos; (3) outcome scores for the care recipient clinical assessments; (4) outcome scores for caregiver self-assessments; and (5) date and time when a caregiver entered a reminder.

### Data Analysis

Interview data were entered into an SPSS (IBM SPSS Statistics Developer) database. Data management and analyses were conducted in Stata (version 17) [41]. Because of missing data, the sample was restricted to participants with observations at baseline and the 3-month follow-up, yielding a final sample of 13. Descriptive statistics and bivariate analyses including analysis of variance and paired-sample 1-tailed *t* tests.

### Ethical Considerations

The design and procedures for this study received approval from the University of Alabama Institutional Review Board, protocol #19-06-2486, and other involved institutions approved reliance for this protocol approval. Participants were required to electronically sign a consent form using Docusign (DocuSign Inc); they received US $25 for each interview they completed and the data were de-identified.

## Results

A total of 21 caregivers initially enrolled in the study (n=13 at the Frank C. and Lynn Scaduto MIND Institute at Miami Jewish Health and n=8 at the University of Alabama at Birmingham). For one caregiver, interviews were conducted in Spanish. Overall, participants were most likely to be a non-Latina white woman caring for a parent or spouse, though the sample was diverse, with 14.3% identifying as African American or Afro Caribbean caregivers and 23.8% identifying Latino/a. Caregivers tended to have moderate to higher incomes and more than two-thirds (68.2%) had a bachelor’s or graduate degree. Most participants lived in urban (40.9%) or suburban (50%) communities. Table 1 provides detailed information about the demographics of participating caregivers. A bivariate analysis with outcome variables demonstrated that burden was associated with caregiver income at baseline, but this relationship disappeared at 3-month follow-up (*t*_20_=8.42, *P*<.001). No other relationships were found between demographic and outcome variables.

Of the 21 caregiver participants completing the PHQ-4 at baseline, 66.6% (n=7) were classified as having normal psychological distress, 47.5% (n=10) mild, 9.5% (n=2) moderate, and 9.5% (n=2) severe distress. All 21 caregivers reported burden, where 2 (9.6%) scored in the mild range, 5 (23.9%) scored in the moderate range, and the remainder (n=14, 66.5%) reported high levels of burden.

Baseline data also demonstrated that many caregivers who enrolled in the project had high health literacy and were active in care management. The average score for caregivers on the ADKS at baseline was 25.71 (SD 2.31, range 21‐28), indicating that many caregivers in the sample were knowledgeable (accuracy approaching 90%) about dementia when they enrolled in the study. In terms of communication with providers, on average caregivers reported that they *fairly often* prepare a list of questions for providers (mean 3.67, SD 1.35), *fairly often* discuss any personal problems that may be related to their loved one’s condition (mean 3.43, SD 1.66) and *very often* ask questions about the things they want to know and the things they do not understand about their loved one’s treatment (mean 4.62, SD 0.74).

A missingness analysis was conducted to determine if there were any relationships between participant demographics and attrition at follow up. An independent samples 1-tailed *t* test examining the mean differences between those who completed the Zarit and PHQ-4 at baseline and the 3-month follow-up with those who didn’t complete them at follow-up did not show any significant differences in their scores for either outcome variable. However, a *χ*^2^ test with Fisher exact test was conducted to examine the demographic characteristics of those who completed both baseline and 3-month follow-up assessments on the PHQ-4 total score, anxiety, and depression. It showed that those who identified as Hispanic or Latino/a were more likely to be lost at follow-up than those who did not. This result suggests that the sample might not be entirely representative, as certain demographic groups (in this case, Hispanic participants) might be underrepresented in the group with complete data.

**Table 1.** Demographic characteristics of the caregiver participants and bivariate analysis with Zarit at baseline.

Characteristics	Participants, n (%)	Baseline (N=21)		
		Mean (SD)	*t*/*F* test (*df*)	*P* value
**Gender**			–1.26 (19)	.22
Female	18 (85.7)	21.22 (8.14)		
Male	3 (14.3)	14.67 (10.07)
Average age (range 43‐80 years)	—^a^	65.32 (10.66)		
**Race**			–0.66 (19)	.52
White or Caucasian	18 (85.7)	19.78 (8.73)		
African American or Afro-Caribbean	3 (14.3)	23.33 (7.51)
**Ethnicity**	0.09 (19)	.93
Hispanic or Latino/a	5 (23.8)	21.26 (6.22)
Not Hispanic or Latino/a	16 (76.2)	23.33 (14.83)
**Household income (US $)**			8.42 (19)	<.001
Less than 20,000	1 (4.8)	37.00 (0)		
20,000-34,999	2 (9.5)	5 (1)
35,000-49,999	3 (14.3)	22 (4)
50,000-74,999	3 (14.3)	29 (4)
75,000-99,000	5 (23.8)	17 (3)
100,000 or greater	7 (33.3)	20 (6)
**Educational attainment**			0.56 (20)	.65
Some college, no degree	3 (13.6)	14.00 (5.00)		
Associate’s degree	4 (18.2)	22.00 (9.00)
Bachelor’s degree	8 (36.4)	21.00 (11.00)
Graduate or professional degree	7 (31.8)	21.00 (7.00)
**Community setting**			—	—
Rural	2 (9.1)	—		
Suburban	11 (50)	—
Urban	9 (40.9)	—
**Relationship to care recipient**			—	—
Wife	11 (50)			
Husband	3 (13.6)
Daughter	5 (22.7)
Daughter-in-law	3 (13.6)
Average length of time caregiving (range)	5.45 (0.5- 18) years	—	—	—
**Living situation**			—	—
Lives with care recipient	18 (77.3)	—		
Does not live with care recipient	3 (22.7)	—
**Self-rated computer skills (n=21)**			—	—
Beginner	1 (4.7)	—		
Average	12 (57.1)	—
Advanced	7 (33.3)	—
Expert	1 (4.7)	—

a Not applicable.

### Usage Data Findings

**Figure 3.**
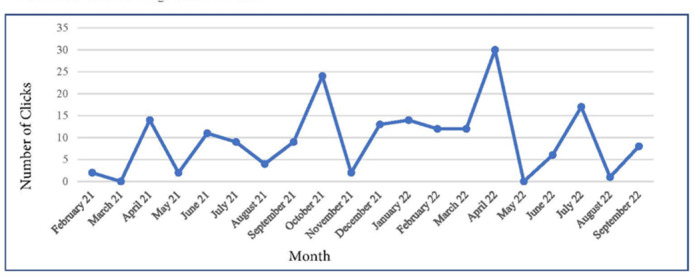
CareHeroes usage data over time.

Usage data were collected between February 2021 and September 2022. Figure 3 shows the user activity for *CareHeroes* over time. It should be noted that recruitment took place over a 14-month period, and throughout the project, enrollment ranged from 2 to 12 caregivers at any given time point. However, enrollment peaked between December 2021 and August 2022, which is reflected in the data. Overall, there were 169 actions that caregivers made on *CareHeroes* (eg, link clicks). Participants most often accessed: the chatbot (44 sessions), resource links (30 clicks), the Zarit Caregiver Burden Inventory (22 submissions), and the Revised Memory and Behavior Problem Checklist (19 submissions). Fewer caregivers access the reminder feature (8 entries) and PHQ-2 assessment (13 submissions).

For the chatbot data, caregivers accessed the chatbot 44 times over the course of the study. The most common topics that caregivers explored on the chatbot were how to manage the depression of the care recipient (n=5 total chats), sleep problems experienced by the care recipient (n=4 total chats), and about living wills (n=4 total chats).

### Interview Outcome Data

The outcome measure for knowledge about Alzheimer disease was only scheduled to be assessed at baseline and at the 12-month follow-up. The sample of caregivers who completed 12-month interviews was too small to evaluate this outcome. However, there was enough data to assess outcomes for burden, depression, and anxiety.

Among the 21 caregivers with baseline data, 12 completed the PHQ-4 and 13 completed the Zarit CBI at the 3-month follow-up. At follow-up, it was found that there was a decrease in total scores for the PHQ-4, though this relationship was not statistically significant (*t*_11_=1.56, *P*=.15). However, when an 1-tailed *t* test was performed with the separate subscales for the PHQ-4, it was found that caregivers reported significantly lower levels of depression at the 3-month follow-up (mean 1.08, SD 1.08) than they did at baseline (mean 1.67, SD 1.48; *t*_11_=2.03; *P*=.03; see Table 2). There was no difference in scores for anxiety during the same time period (*t*_11_=1.0667, *P*=.15).

**Table 2.** Paired samples 1-tailed *t* test for burden, anxiety, and depression.

	Mean (SD)	*t* test (*df*)	*P* value
**Zarit (n=13)**		–0.07 (12)	.95
Baseline	21.85 (6.38)		
3-month	21.92 (7.89)		
**2 Anxiety items**^**a**^ **(n=12)**		1.0667 (12)	.15
Baseline	2.25 (1.48)		
3 Months	1.75 (1.29)		
**2 Depression items**^**a**^ **(n=12)**		2.03 (12)	.03
Baseline	1.67 (1.32)		
3 Months	1.08 (1.08)		

a Patient Health Questionnaire for Depression and Anxiety (PHQ-4) was administered, and from the PHQ-4 items, the 2-item measure of depression (PHQ-2) and the 2-item measure of anxiety (the Generalized Anxiety Disorder-2 scale [GAD-2]) were analyzed.

## Discussion

### Principal Findings

Over the past decade, the development and research of new technologies to support people living with dementia and their caregivers has increased significantly. However, little remains known about how such technologies can be integrated into clinical care to facilitate caregiver support and secure clinical information sharing. In this pilot study, we were able to demonstrate that caregivers did use *CareHeroes* for education and support. We also demonstrated that new HIT can be integrated into care for secure sharing of patient information in real time. Although outcome data suggests that there was a decrease in caregiver depression at the 3-month follow-up, more research is needed to fully assess the potential impact of using *CareHeroes* on psychosocial outcomes.

### Potential of Chatbots for Caregiver Support

Overall, this pilot study recruited a smaller sample than initially planned, and attrition of caregivers in the study was high, which created limits to assessing outcome variables. However, user data indicated that caregivers accessed a variety of features on the app, though the most used feature on the app was the educational chatbot. This was notable because very few chatbots have been developed to support people living with dementia and their caregivers [17]. Chatbots have grown in popularity over the past decade and have become a familiar technology to many through popular chatbots like Apple’s Siri and Amazon’s Alexa [42]. Chatbots offer benefits over other types of information technologies for health education and support in that they may be programmed so they are tailored to the user’s needs. Also, recent advances in chatbots based on large language models, such as ChatGPT, have the potential to significantly improve the responsiveness to caregivers’ support needs.

In a recent study, ChatGPT and Google were evaluated on their ability to respond to common questions that caregivers have [43]. ChatGPT was found to produce results that were more closely aligned with what the question was asking, though it did not provide sources or how old the information was. Both ChatGPT and Google were found to provide responses that were written at a higher reading level than what is recommended for general health education. Similar to other AI chatbot tools, the bot’s responses to the user’s input require regular training. As a result, the quality and availability of information is dependent on how often the chatbot is trained and the content used to train it. As earlier discussed, the chatbot in the *CareHeroes* app is programmed with expert feedback to common questions that caregivers have about dementia and caregiving [35]. Hence, while a Google search of similar questions may result in an overwhelmingly large number of search results that are irrelevant, the *CareHeroes* chatbot is able to provide more direct responses to the questions caregivers often have. In other health settings, it has been found that chatbots can be an effective way of promoting health education, treatment management, and moral support [44,45]. It has also been suggested that chatbots may be an ideal platform for collecting patient-related information [46]. Moving forward, the implementation of *CareHeroes* may be more successful if more features were offered in a chatbot format.

### Limitations and Future Research

Like many studies during this time period, the COVID-19 pandemic posed significant challenges to recruiting and retaining caregivers. Initially, the research team thought the increase in telehealth during the pandemic would possibly lead to more caregivers wanting to participate in the study. However, anecdotally, many caregivers reported to the clinic staff that they were overwhelmed by the pandemic and its associated increased need for telecommunication. In such cases, caregivers expressed that learning another new technology was too burdensome. Among the caregivers who enrolled in the study, some reported to interviewers at the 12-month follow-up that they wished they had used the app more because they thought the features would have helped them, but it was difficult to fit using the app within their daily care activities during the pandemic. Similarly, the clinic staff reported that understaffing during the pandemic made it difficult for them to log onto *CareHeroes* regularly. While this pilot study provides direction for future research, more research is needed under more typical circumstances to generate strong evidence about the potential of HIT as an adjunct to clinical care for people living with dementia and their caregivers.

One challenge in the study stemming from the pandemic was that the research team was unable to meet with caregivers in person when they enrolled in the study to orient them with the technology when it was first introduced. This may have impacted their familiarity with using the app, since they were only given instructions over the telephone or Zoom. Future research should identify best practices in training caregivers to use new HIT so that they gain the maximum benefit that the technology has to offer. Similarly, how best to provide ongoing support to caregivers should be identified. For example, examining the effectiveness of embedding instructional videos in the software design on maintaining caregiver engagement with the technology. In this study, caregivers were provided with a helpline that they could call when they encountered a problem with *CareHeroes*. However, it is possible that some caregivers experienced challenges that they did not report to the helpline and stopped using the app when they could not resolve the issue themselves.

Another limitation to this study is that the caregiver participants who enrolled in the study were very experienced and very knowledgeable about ADRD at baseline, which may be associated with their access to specialized memory care, as opposed to primary care. It is suspected that newer caregivers would interact with and learn from the *CareHeroes* app differently. However, the findings from the study’s implementation evaluation and outcome assessment provide future researchers with insight on developing tech-focused interventions.

In addition, future studies should also examine how to better integrate new information technologies into clinical care so that they are easily adopted by providers. For example, designing new technologies so that they can be integrated into existing secure systems, like electronic medical records, rather than offering additional programs that require new log-ins can minimize the burden on providers who have to navigate multiple secure software programs on a regular basis. Similarly, more research is needed on technologies that can encourage triadic communication between people living with dementia, their caregivers, and providers. While *CareHeroes* was designed to empower caregivers of patients with dementia, the research team recognizes that more should be done to empower these patients to promote person-centered care.

### Conclusion

There has been growing interest in the development of technologies that support people living with dementia and their caregivers. While the features and functions of new technologies will impact their adoption, this paper highlights the importance of implementation planning in order to maximize success in tech adoption, particularly by caregivers. While these findings may provide future direction for researchers involved in developing technologies for dementia care and caregiving, they are also relevant to commercial technology developers who are interested in integrating similar technologies within real-world contexts. Though this research experienced several limitations stemming from the COVID-19 pandemic, the research team credits the early work done to overcoming these challenges and successfully implementing *CareHeroes*.

## Abbreviations

ADKS: Alzheimer’s Disease Knowledge Scale
ADRD: Alzheimer disease and related dementias
AI: artificial intelligence
GAD-2: Generalized Anxiety Disorder 2 item
HIPAA: Health Insurance Portability and Accountability Act
HIT: health IT
PHQ: Patient Health Questionnaire for Depression and Anxiety
